# Genome-wide identification and expression analysis of the *regulator of chromosome condensation 1* gene family in wheat (*Triticum aestivum* L.)

**DOI:** 10.3389/fpls.2023.1124905

**Published:** 2023-02-24

**Authors:** Xia An, Shuqi Zhao, Xiahong Luo, Changli Chen, Tingting Liu, Wenlue Li, Lina Zou, Chendong Sun

**Affiliations:** ^1^ Zhejiang Xiaoshan Institute of Cotton and Bast Fiber Crops, Zhejiang Institute of Landscape Plants and Flowers, Zhejiang Academy of Agricultural Sciences, Hangzhou, China; ^2^ Cotton and Wheat Research Institute, Huanggang Academy of Agricultural Sciences, Huanggang, China; ^3^ The Institute of Horticulture, Zhejiang Academy of Agricultural Sciences, Hangzhou, Zhejiang, China

**Keywords:** wheat, *regulator of chromosome condensation 1* (*RCC1)*, gene family, abiotic stress, expression analysis

## Abstract

Wheat (*Triticum aestivum* L., 2*n* = 6*x* = 42, AABBDD) is the world’s most widely cultivated crop and an important staple food for humans, accounting for one-fifth of calories consumed. Proteins encoded by the *regulator of chromosome condensation 1* (*RCC1*) are highly conserved among eukaryotes and consist of seven repeated domains that fold into a seven-bladed propeller structure. In this study, a total of 76 *RCC1* genes of bread wheat were identified *via* a genome-wide search, and their phylogenetic relationship, gene structure, protein-conserved domain, chromosome localization, conserved motif, and transcription factor binding sites were systematically analyzed using the bioinformatics approach to indicate the evolutionary and functional features of these genes. The expression patterns of 76 *TaRCC1* family genes in wheat under various stresses were further analyzed, and RT-PCR verified that *RCC1-3A* (*TraesCS3A02G362800*), *RCC1-3B* (*TraesCS3B02G395200*), and *RCC1-3D* (*TraesCS3D02G35650*) were significantly induced by salt, cold, and drought stresses. Additionally, the co-expression network analysis and binding site prediction suggested that *Myb-7B* (*TraesCS7B02G188000*) and *Myb-7D* (*TraesCS7D02G295400*) may bind to the promoter of RCC1-3A/3B and upregulate their expression in response to abiotic stresses in wheat. The results have furthered our understanding of the wheat *RCC1* family members and will provide important information for subsequent studies and the use of *RCC1* genes in wheat.

## 1 Introduction

The *regulator of chromosome condensation 1* (*RCC1*) genes encode proteins whose sequence is highly conserved among eukaryotes and consists of seven repeated domains that fold into a seven-bladed propeller structure **(** Renault et al., 1998 **)**. RCC1 proteins in mammals act as the guanosine nucleotide exchange factors (GEFs) for a GTPase well known as Ras-related nuclear protein (Ran) and are involved in diverse biological processes, such as spindle assembly, nuclear membrane formation, and nucleocytoplasmic transport during mitosis **(** Bischoff and Ponstingl, 1991; Hutchins et al., 2004; Li and Zheng, 2004; Chen et al., 2007; Terry et al., 2007 **)**. RCC1 proteins are implicated in the initiation and progression of a variety of cancers by promoting nuclear entry and accumulation of β-catenin **(** Brabletz et al., 2001; Polakis, 2007; Dominguez et al., 2009 **)**. Interestingly, several studies have reported that another class of fungal protein, latcripin, which contains the RCC1 domain, can effectively promote the apoptosis of cancer cells **(** Liu et al., 2012; Ann et al., 2014; Tian et al., 2016; Wang et al., 2016 **)**.

*RCC1* family genes are also present in plants. However, a few plant *RCC1* genes have only been identified successively in the last decade or so. RCC1 family proteins in plants can be divided into two major groups: single-domain proteins (containing only a single RCC1 repeat domain) and multi-domain proteins (containing other domains in addition to the RCC1 repeat domain) **(** Sun et al., 2022 **)**. Single-domain RCC1 family proteins have been identified in both animals and plants, while PRAF (PH, RCC1, and FYVE) proteins, a class of typical multi-domain RCC1 family proteins, are unique to plants and contain four distinctive domains: two lipid‐binding domains, including pleckstrin homology (PH) and FYVE (Fab1, YOTB, Vac 1, and EEA1) zinc‐finger domains, the RCC1 (seven repeats of the regulator of chromosome condensation 1) or alpha‐tubulin suppressor domain1 (ATS1) motif, and a C‐terminal BRX/DZC (brevis radix/disease resistance, zinc finger, chromosome condensation) domain **(** Sun et al., 2022 **)**. In *Arabidopsis*, there are 24 putative proteins containing the RCC1-like domains, but only five have been functionally studied **(** Brown et al., 2005; Kuhn et al., 2011; Ji et al., 2015; Su et al., 2017; Ji et al., 2019; Duarte et al., 2021 **)**. *Arabidopsis* UV RESISTANCE LOCUS 8 (UVR8) is the first plant RCC1 family member to be identified as the only UV receptor in plants **(** Kliebenstein et al., 2002; Rizzini et al., 2011; Christie et al., 2012; Wu et al., 2012; Jenkins, 2017 **)**. Upon absorbing UV-B radiation, UVR8 immediately switches from homodimer to monomer and then accumulates in the nucleus through interaction with constitutive photomorphogenic 1 (COP1), triggering a UV-B cascade, thus regulating the expression of downstream genes and plant responses to UV-B **(** Brown et al., 2005; Kaiserli and Jenkins, 2007; Favory et al., 2009; Rizzini et al., 2011; Yin et al., 2016 **)**. RCC1/UVR8/GEF-like 3 (RUG3), another *RCC1* family protein, interacts with ataxia–telangiectasia mutant (ATM) protein in mitochondria to synergistically regulate the splicing of *nad2* mRNA and its complex function, which is necessary for reactive oxygen species homeostasis and plant development **(** Kuhn et al., 2011; Su et al., 2017 **)**. The third characteristic protein of the *RCC1* family protein in *Arabidopsis* is tolerant to chilling and freezing 1 (TCF1), which is located in the nuclear genome and regulates plant cold adaptation and tolerance through a chromatin-based regulation mechanism **(** Ji et al., 2015 **)**. Under cold stress, *TCF1* is upregulated rapidly and affects the expression of the *blue copper-binding protein* (*BCB*), which regulates lignin biosynthesis and subsequent cell wall remodeling **(** Ji et al., 2015 **)**. Another *RCC1* family protein, sensitive to ABA 1 (SAB1), can bind to the promoter of *abscisic acid-insensitive 5* (*ABI5*) and inhibit its expression by increasing the level of histone H3K27me2 in the *ABI5* promoter, thus negatively regulating the seed germination process **(** Ji et al., 2019 **)**. Recent research focused on *RCC1* genes in *Arabidopsis* revealed that another RCC1 family protein, PROTON1, regulates rosette leaf growth in response to nitrogen availability **(** Duarte et al., 2021 **)**. In addition to *Arabidopsis*, a number of *RCC1* genes have successively been identified in other plants. In cotton, two *RCC1* family genes showed crucial roles in salt tolerance **(** Liu et al., 2019 **)**. GmTCF1a responds specifically to cold stress and positively regulates cold tolerance in soybean **(** Dong et al., 2021 **)**. In maize, the RCC1 family protein Dek47 can influence the assembly of the mitochondrial complex and maize seed development by regulating the splicing of the nad2 transcript (Cao et al., 2021). SaRCC1, an RCC1 family protein in *Spartina alterniflora*, was found to negatively regulate salt tolerance in plants by using a heterologous expression assay in *Arabidopsis* **(** Li et al., 2022 **)**. In *Medicago truncatula*, PRAF protein MtZR1 (belonging to the multi-domain RCC1 family proteins) is a cytomembrane‐ and nuclear‐located protein that plays a key role in root development and symbiotic root nodules **(** Hopkins et al., 2014 **)**. In rice, another PRAF family protein, OsRLR4, alters OsAUX1 promoter histone H3K4me3 levels by recruiting the histone methyltransferase OsTrx1, which promotes *OsAUX1* expression, alters auxin accumulation in root tips, and ultimately affects the root apical meristem (RAM) activity **(** Sun et al., 2022 **)**.

Wheat (*Triticum aestivum* L., 2*n* = 6*x* = 42, AABBDD) is the world’s most widely cultivated crop and an important staple food for humans, accounting for one-fifth of calories consumed **(** International Wheat Genome Sequencing, C 2018 **)**. No study on the RCC1 domain proteins in wheat has been reported, mainly because of the later release of the genome than in other species. Fortunately, with the release of the high-quality reference genome and annotation of the Chinese Spring (CS, a bread wheat cultivar from China) by the Wheat Genome Sequencing Consortium (IWGSC) **(** International Wheat Genome Sequencing, C 2018 **)**, rapid and systematic methods for understanding wheat genomics and genetics have been rapidly developed. In the present study, a total of 76 *RCC1* genes of bread wheat were firstly identified with a genome-wide scan on the latest released wheat genome, and then a systematical analysis, including the gene phylogenetic relationship, gene structure, protein-conserved domain, chromosome localization, conserved motif, and transcription factor binding sites, was performed for these genes to indicate their evolutionary and functional features. The tissue-specific and stress-induced expression of these genes was also examined using public RNA-seq data and real-time quantitative PCR (qRT-PCR). The results have furthered our understanding of the wheat *RCC1* family members and will provide important information for subsequent studies and use of *RCC1* genes in wheat.

## 2 Materials and methods

### 2.1 Identification of *RCC1s* in bread wheat, emmer wheat, and *Aegilops tauschii*

The hidden Markov model (HMM) profile of the *RCC1* gene family (PF00415) in PFAM (http://pfam.xfam.org/) was downloaded and used to identify the *RCC1* genes in the local protein database of bread wheat, emmer wheat (*Triticum dicoccoides*, 2*n* = 4*x* = 28, AABB) and *Aegilops tauschii* (2*n* = 2*x* = 14, DD) (downloaded from Ensembl Plants, http://plants.ensembl.org/index.html) with the hmmsearch tool of HMMER3.1 software (HMMER 3.1; http://hmmer.org/). To avoid missing *RCC1* family members, an aligned file of a high-quality protein set (*E* value < 1 × 10^−20^) in MEGA X software **(** Kumar et al., 2018 **)** was used to reconstruct the new HMM profile, which was used as the query to search all the *RCC1* members (*E* value < 0.01) in all bread wheat, emmer wheat, and *Aegilops tauschii* proteins, respectively. All the detected protein sequences were submitted to the PFAM (http://pfam.xfam.org/), SMART domain search (http://smart.embl.de/smart/batch.pl) **(** Letunic et al., 2021 **)**, and NCBI Batch CD-search database (https://www.ncbi.nlm.nih.gov/Structure/bwrpsb/bwrpsb.cgi) to confirm the structural integrity of the RCC1 domain **(** Marchler-Bauer et al., 2005 **)**. The non-redundant, verified genes encoding proteins with RCC1 domains were assigned as members of the *RCC1* gene family.

### 2.2 Conserved sequence and phylogenetic analysis

Multiple alignments of the conserved RCC1 protein sequences of bread wheat, emmer wheat, and *Aegilops tauschii* were performed using Clustal Omega **(** Sievers et al., 2011 **)** using default parameters, and a phylogenetic tree was constructed using a maximum-likelihood method with 1,000 bootstrap replications in the RaxML_NG software **(** Kozlov et al., 2019 **)**. Figtree 1.4.4 (http://tree.bio.ed.ac.uk/software/figtree/) was used to visualize and optimize the phylogenetic tree.

### 2.3 Chromosomal locations and synteny analysis

The *RCC1* gene loci of wheat and its related genome donors were extracted from the corresponding annotated gff3 file (downloaded from Ensembl Plants, http://plants.ensembl.org/index.html) using a perl script. The Multiple Collinearity Scan toolkit (MCScanX) was used to analyze the gene collinearity among wheat, emmer wheat, and *Aegilops tauschii* with the default parameters **(** Kozlov et al., 2019 **)**.

Homolog analysis of *RCC1* genes among the A, B, and D genomes of wheat was performed based on the aligned result. The chromosomal distribution and collinearity of *RCC1* genes among the wheat and its donors and of the homoeologous *RCC1* genes among A, B, and D genomes were visualized by the circle package in R **(** Gu et al., 2014 **)**.

### 2.4 Characterization of gene structure, protein domains, and motifs

Clustal Omega **(** Sievers et al., 2011 **)** was used to analyze RCC1 protein sequences of wheat, and RaxML_NG **(** Kozlov et al., 2019 **)** was used to construct a phylogenetic tree *via* a maximum-likelihood method with 1000 bootstrap replications. The domains of the *RCC1* gene family in wheat were verified by the SMART domain search (http://smart.embl.de/smart/batch.pl) **(** Letunic et al., 2021 **)** and the NCBI Batch CD-search database (https://www.ncbi.nlm.nih.gov/Structure/bwrpsb/bwrpsb.cgi) **(** Marchler-Bauer et al., 2005 **)**. The conserved motifs of the *RCC1* gene family in wheat were determined by the online Multiple Em for Motif Elicitation (MEME) suite program (http://meme-suite.org) **(** Bailey et al., 2006 **)**. The software TBtools **(** Chen et al., 2020 **)** was used to visualize the gene structure, protein domains, and motifs of the *RCC1* genes according to the annotated GFF files, the genome sequence of wheat, and the protein domain file from the SMART domain search database, as well as the motif result files from the MEME suite.

### 2.5 Identification of putative cis-acting regulatory elements

The promoter sequences (2-kb upstream) of the *TaRCC1* genes were extracted from the wheat reference genome (IWGSC RefSeq v1.1, International Wheat Genome Sequencing, C 2018) using the GTF/GFF3 Sequences Extract function of TBtools **(** Chen et al., 2020 **)**, and their potential cis-acting elements were predicted by submitting to PlantCARE (http://bioinformatics.psb.ugent.be/webtools/plantcare/html/).

### 2.6 Expression profiles of *TaRCC1* genes

To analyze the tissue-specific and stress-induced expression of *TaRCC1* genes, the RNA-seq expression data of five publicly available studies **(** International Wheat Genome Sequencing, C 2014; Zhang et al., 2014; Li et al., 2015; Liu et al., 2015; Zhang et al., 2016 **)** were obtained from expVIP Wheat Expression Browser (http://www.wheat-expression.com/) **(** Ramirez-Gonzalez et al., 2018 **)** and Triticeae Multi-omics Center (http://202.194.139.32/expression/index.html) and then visualized using the pheatmap package of R software.

### 2.7 Prediction of transcription factors regulating the expression of *TaRCC1* genes

A KnetMiner web application for wheat (https://knetminer.com/Triticum_aestivum/, HassaniPak and Keywan, 2017) was used to search gene-evidence networks extracted from the knowledge network and predict the transcription factors for the three *TaRCC1* genes. The expression pattern of the predicted transcription factors was detected by qRT-PCR.

### 2.8 Subcellular localization of *TaRCC1*

The full-length coding DNA sequences (CDS) of RCC1-3A (TraesCS3A02G362800), RCC1-3B (TraesCS3B02G395200), RCC1-3D (TraesCS3D02G356500), Myb-7B (TraesCS7B02G188000), and Myb-7D (TraesCS7D02G295400) were inserted into pCambia1300-35S-GFP, creating RCC1-3A::GFP, RCC1-3B::GFP, RCC1-3D::GFP, Myb-7B::GFP, and Myb-7D::GFP fusion vectors. The recombinant plasmids were mixed with the nuclear marker NLS-mCherry and transfected into wheat mesophyll protoplasts as previously described by Yoo et al. (2007). The transfection mixture was induced by PEG-Ca^2+^, and the protoplasts were cultured for 12 h at 25°C. The protoplasts were observed and photographed with a fluorescence microscope (Zeiss Imager A2, Germany).

### 2.9 Plant materials and treatments

The bread wheat cultivar, CS, was grown in a greenhouse with controlled conditions of 26°C/14 h light and 20°C/10 h dark. Three different treatments were applied, namely salt stress, cold, and drought stress induced by polyethylene glycol (PEG). During the two-leaf stage, seedlings were treated with Hoagland liquid medium containing 200 mM NaCl for 1, 3, and 6 h (salt stress), 4°C for 1, 3, and 6 h (cold stress), and 20% PEG4000 for 1, 3, and 6 h (drought stress). Seedlings grown in a normal environment without treatment were set as the control. Three biological replicates were set for all the trials.

### 2.10 Total RNA isolation and gene expression by quantitative real-time PCR

The total RNA of plant materials was extracted using the RNAprep Pure Plant Kit (polysaccharide- and polyphenolic-rich) (TIANGEN, Beijing, China), following the manufacturer’s instructions. A NanoDrop One spectrophotometer (NanoDrop Technologies, Wilmington, DE, USA) and agarose gel electrophoresis were used to assess RNA quantity and purity. Complementary DNA (cDNA) was synthesized using Reverse Transcriptase M-MLV (Takara, Beijing, China) according to the manufacturer’s instructions. qRT-PCR was performed using a 7300 Real-Time PCR System (Applied Biosystems, Foster City, CA, USA) according to the supplier’s instructions. A total of 6 L of DNase/RNase-free water, 11 μl of TB Green Real-Time PCR master mix, 2 μl of diluted cDNA product, and 1 μl of gene-specific primer was added to each reaction mixture. Three biological replicates were used for each tissue and three technical repeats for each biological replicate. The thermal cycle was set as follows: denaturing at 95°C for 30 s, then denaturing at 95°C for 15 s, and annealing and elongating at 58°C for 30 s with 45 cycles. The GAPDH gene was used as an internal reference for the normalization of the expression of the *TaRCC1* genes. The relative expression levels were calculated using the 2^−ΔΔCt^ method.

## 3 Results

### 3.1 Identification of *RCC1* genes in bread wheat, emmer wheat, and *Aegilops tauschii*

**Figure 1:**
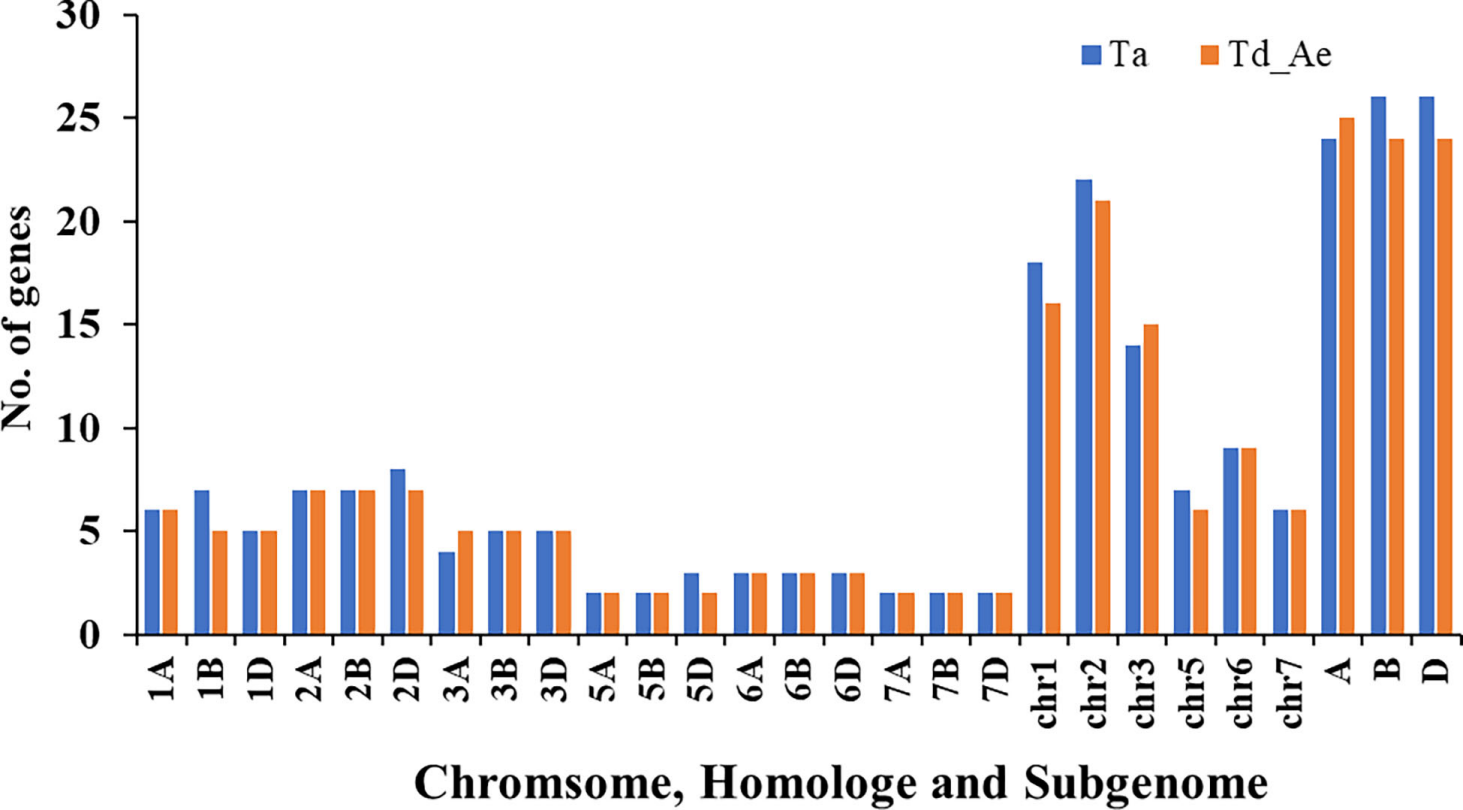
Distribution of RCC1s on chromosomes, different homoeologous groups, and sub-genomes in bread wheat, emmer wheat, and *Aegilops tauschii*.

To identify *RCC1* genes in bread wheat, emmer wheat, and *Aegilops tauschii*, a genome-wide search was performed by local BLASTP using HMM profiles. In total, 149 *RCC1* genes, comprising 76 *TaRCC1s*, 49 *TdRCC1s*, and 24 *AetRCC1*, were identified and verified by detecting the *RCC1*-conserved domain *via* the Pfam (http://pfam.xfam.org/), SMART domain search (http://smart.embl.de/smart/batch.pl), and NCBI Batch CD-search database (https://www.ncbi.nlm.nih.gov/Structure/bwrpsb/bwrpsb.cgi). The details of the identified *RCC1* genes are listed in **Supplementary Table S1**. The distribution of the *RCC1* genes on chromosomes, different homoeologous groups, and sub-genomes was determined (**Figure 1**). Emmer wheat (AABB) and *Aegilops tauschii* (DD) are two genome donors of bread wheat; thus, the *RCC1* genes of emmer wheat and *Aegilops tauschii* were integrated together to compare with bread wheat. The number of *RCC1* genes on chromosomes, homoeologous groups, and sub-genomes showed little difference between bread wheat and the combined data for emmer wheat and *Aegilops tauschii.* Most *RCC1* genes were located in homoeologous groups 1, 2, and 3, while no *RCC1s* were detected in homoeologous group 4. Two *RCC1* genes (*TraesCSU02G009000LC* and *TraesCSU02G009100LC*) on ChrUn of bread wheat were certificated to belong to Chr1B through phylogenetic and synteny analysis with the *RCC1s* of emmer wheat and *Aegilops tauschii* as follows.

### 3.2 Phylogenetic analysis of *RCC1* genes

**Figure 2:**
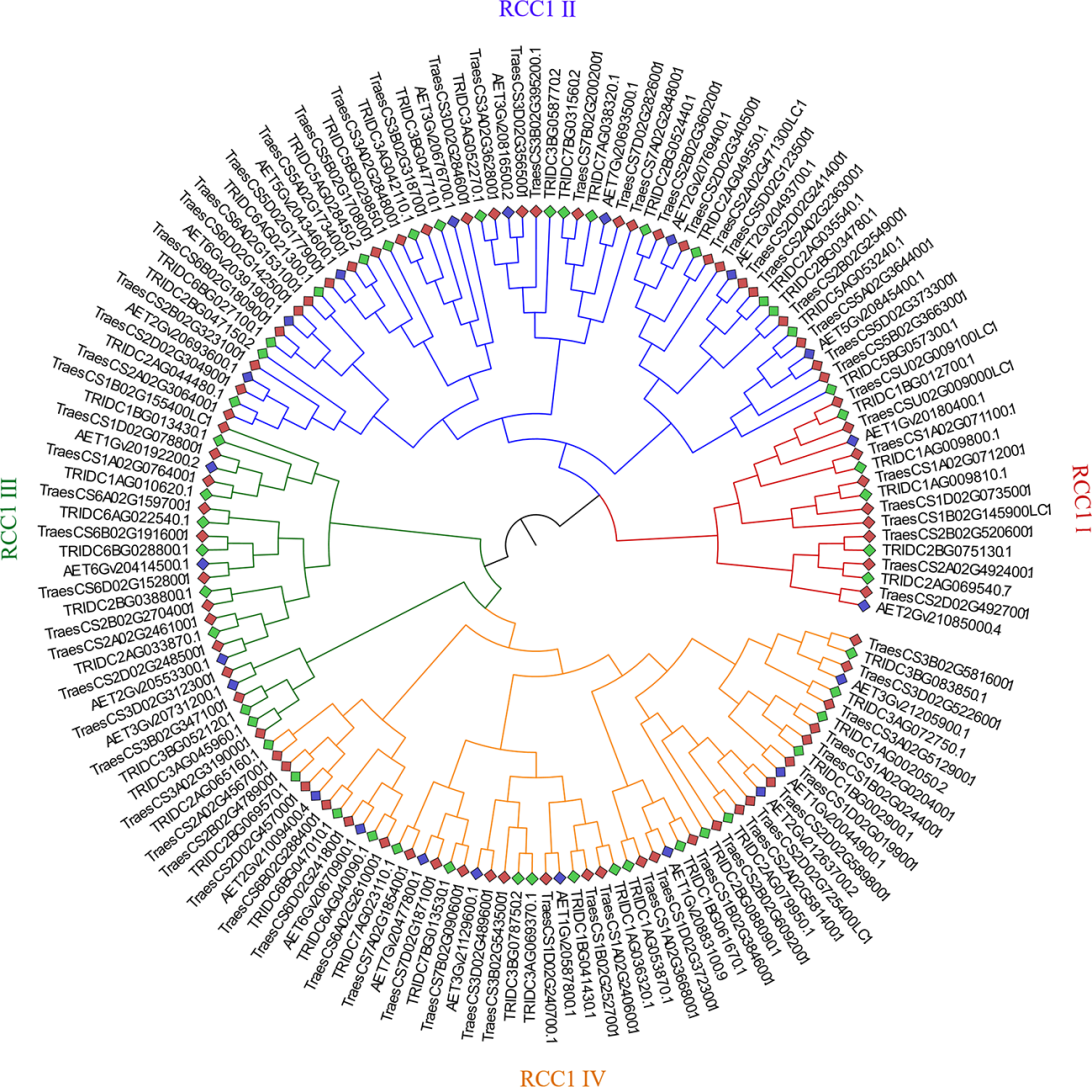
Phylogenetic tree of the RCC1 protein sequences of bread wheat, emmer wheat, and *Aegilops tauschii*. The phylogenetic tree was built using the maximum-likelihood method in the RaxML_NG web server with 1,000 bootstrap replications.

To investigate the phylogenetic relationships and compare the evolutionary relationships of *RCC1* genes among bread wheat, emmer wheat, and *Aegilops tauschii*, a maximum-likelihood phylogenetic tree was constructed using the protein sequences of RCC1s (**Figure 2**). The best-fit model to construct the tree was LG+FC+G8m, and the RCC1s were classified into four subfamilies (sub. I–IV) and named RCC1 I–IV. The RCC1 I, II, III, and IV subfamilies contained 16 (nine for wheat, five for emmer wheat, and two for *Aegilops tauschii*), 55 (28 for wheat, 18 for emmer wheat and nine for *Aegilops tauschii*), 24 (12 for wheat, eight for emmer wheat and four for *Aegilops tauschii*), and 54 *RCC1* genes (27, 18, and nine), respectively. Interestingly, in each subgroup, the number of *RCC1* genes from bread wheat (AABBDD), emmer wheat (AABB), and *Aegilops tauschii* (DD) showed approximately a 3:2:1 ratio, which indicated that the *RCC1* gene is evolutionarily conserved across the three species.

### 3.3 Chromosomal locations and synteny analysis

**Figure 3:**
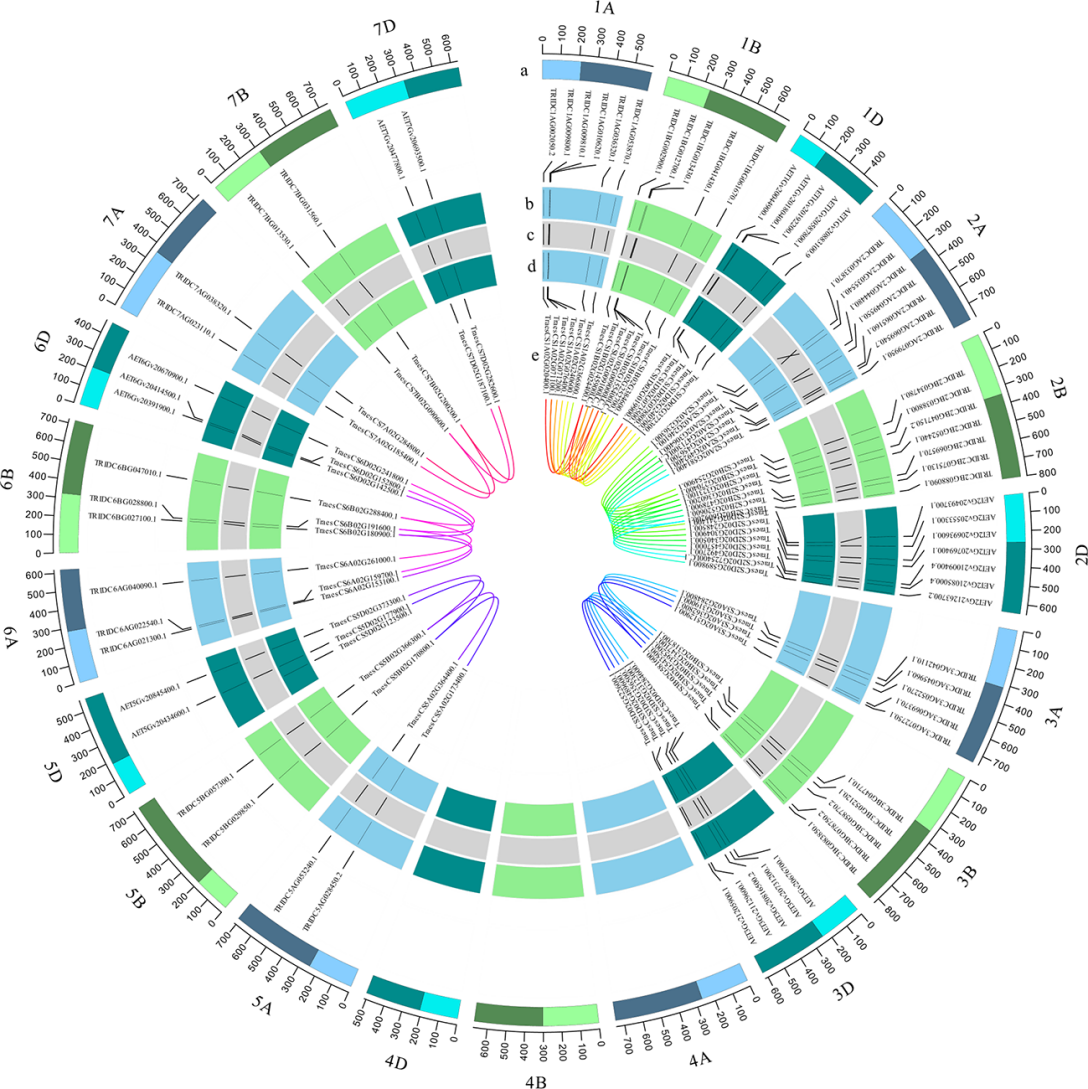
Genomic distribution of *TaRCC1* genes and gene homology analysis in wheat. The tracks toward the center of the circle display (a) the chromosome name and size of wheat (100-Mb tick size; three different colors refer to sub-genomes (a, b, d); the light and dark bars indicate the short and long chromosome arms, respectively); (b) the distribution of *TdRCC1* and *AeRCC1* on chromosomes (the relative positions were adjusted according to the length of the corresponding wheat chromosome; A and B genomes were from emmer wheat; the D genome was from *Aegilops tauschii*). (c) Collinearity of *TaRCC1*, *TdRCC1*, and *AeRCC1*. (d) Genomic distribution of *TaRCC1* genes in the wheat genome. (e) Homoeologous genes among A, B, and D sub-genomes.

There are 76 wheat *TaRCC1* genes mapped to 18 of the 21 wheat chromosomes, except 4A, 4B, and 4D, according to available annotation information of the wheat genome. Synteny analysis showed that most *TdRCC1s* (except *TRIDC3AG069370.1*) and all the *AeRCC1s* were highly collinear with the *TaRCC1s*, and phylogenetic analysis indicated the collinear *RCC1* genes of the three species were clustered together (**Figures 2**, **3**; **Supplementary Table S2**). Homologous gene analysis indicated that, except for *TraesCS5D02G123500.1*, most *TaRCC1* genes were homoeologous to each other among the A, B, and D genomes, and clustered together (**Figures 2**, **3**; **Supplementary Table S3**). It was noteworthy that two unanchored genes, *TraesCSU02G009100LC.1* and *TraesCSU02G009000LC.1* on ChrUn, should be anchored on chromosome 1B for their high collinearity to *TraesCS1D02G073500.1* and *TraesCS1A02G071100.1*; therefore, we adjusted the positions of *TraesCSU02G009100LC.1* and *TraesCSU02G009000LC.1* on chromosome 1B for the visualization of collinearity analysis (**Figure 3**). According to the descriptions, a chromosomal region within 200 kb containing two or more genes is defined as a tandem duplication event. The gene pairs *TraesCS1A02G071100.1/TraesCS1A02G071200.1* and *TraesCSU02G009000LC.1*/*TraesCSU02G009100LC.1* were each clustered into one tandem duplication event region on chromosomes 1A and 1B of bread wheat, respectively; moreover, no homoeologous gene on chromosome 1A was found for the two homoeologous genes *TraesCS3B02G543500.1* and *TraesCS3D02G489600.1*. In general, the *TaRCC1s*, *TdRCC1s*, and *AeRCC1s* on corresponding chromosomes show high collinear with each other according to the synteny analysis (**Figure 3**), and the number of *RCC1* genes on corresponding chromosomes, homoeologous groups, and sub-genomes in bread wheat, emmer wheat, and *Aegilops tauschii* showed approximately a 1:1 ratio, respectively (**Figure 1**), which indicated that *RCC1* genes were highly conserved during the evolution of wheat, and the expansion of RCC1 gene family was mostly due to the genome polyploidization.

### 3.4 Gene structure, protein domains, and motif analysis of *TaRCC1s*

**Figure 4:**
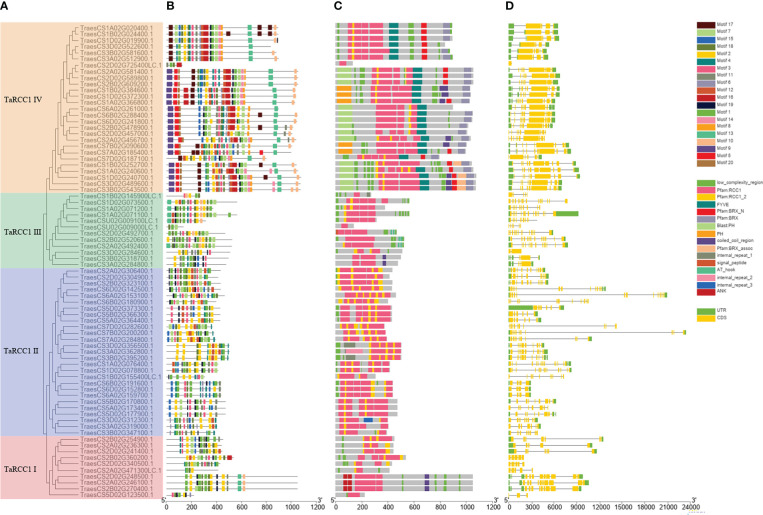
Phylogenetic relationship, conserved motifs, protein-conserved domains, and gene structure analysis of *TaRCC1* genes. **(A)** Phylogenetic tree of 76 TaRCC1 proteins. **(B)** Conserved motifs of TaRCC1 proteins. **(C)** Conserved domains of TaRCC1 proteins; different domains are marked with different colors. **(D)** Exon–intron structures of *TaRCC1* genes: exons are represented by orange boxes, introns are represented by black lines, and the upstream/downstream regions are represented by green boxes.

To further estimate the gene structure, protein-conserved domains, and motifs of wheat *TaRCC1* genes, the full-length protein sequences of 76 *TaRCC1s* were aligned using Clustal Omega, and the phylogenetic tree was constructed using RaxML_NG (**Figure 4A**). The *TaRCC1s* in wheat were classified into four subfamilies named *TaRCC1* I–IV. The *TaRCC1* I, II, and III subfamilies contained 10, 27, and 12 genes, respectively, and carried only RCC1 domain repeats, while the remaining 27 *TaRCC1s* of the *TaRCC1* IV subfamily contained multiple domains, including RCC1 domain repeats and PH or BRX domains (**Figure 4C**). The conserved motifs of the *TaRCC1* genes were determined by the online MEME suite program (http://meme-suite.org): 20 conserved motifs with lengths from 11 to 41 amino acids were detected among the *TaRCC1* genes (**Supplementary Table S4**; **Figure 4B**). *TaRCC1s* in the same cluster shared similar conserved motif compositions, which again indicated that there is high conservation of the *RCC1* gene family sequence in wheat. Despite the similarity of motifs among closely related genes, the size of the gene fragments varied widely (390–23,462 bp), such that the *TraesCS2D02G725400LC* gene fragment was much smaller than *TraesCS7B02G200200*. The gene structure, including the size and number of intron–exon, varies a lot among different *TaRCC1s* (such as the number of exons is from 1 to 17) (**Figure 4D**). It is worth noting that the closely related members, especially homologous genes, showed similar exon–intron structure, and the difference among them was the exon–intron length. The homologous genes *TraesCS7A02G284800*, *TraesCS7B02G200200*, and *TraesCS7D02G282600* were similar in motif, protein domains, and exon–intron structure, while their exon–intron lengths varied greatly (10,997 bp, 23,461bp and 14,257 bp, respectively) (**Figure 4**; **Supplementary Table S1**).

### 3.5 Cis-acting elements in the promoters of *TaRCC1s*

**Figure 5:**
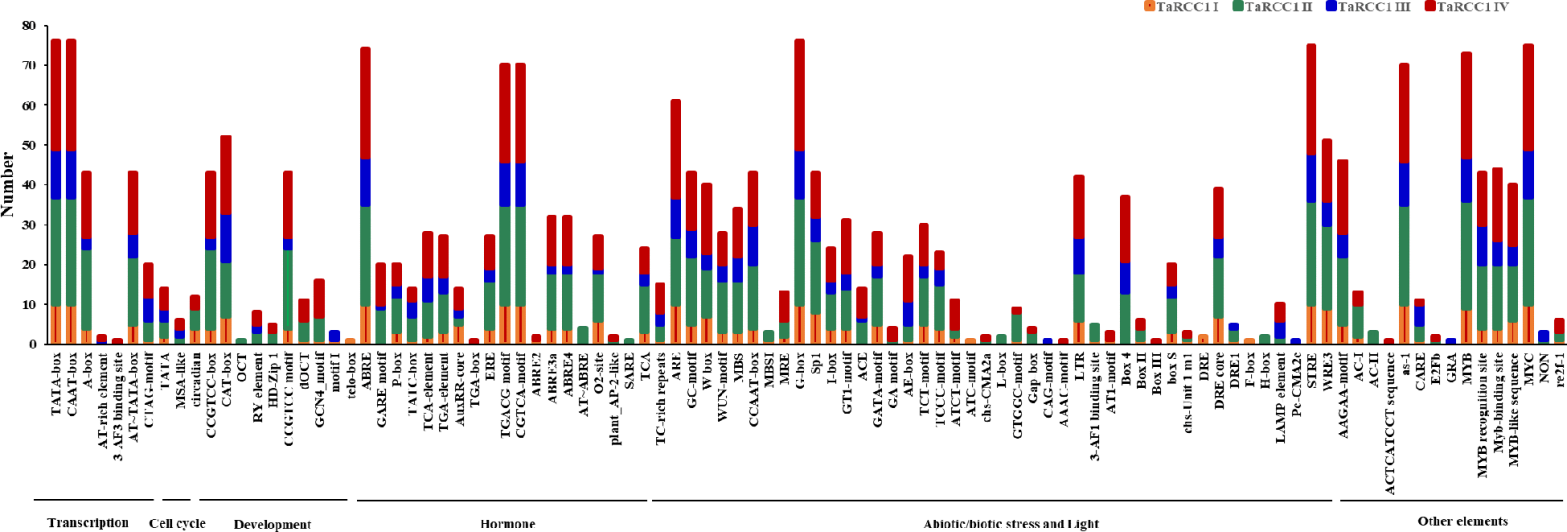
Cis-acting regulatory elements of *TaRCC1* genes. The graph was generated using cis-acting element names and functions of *TaRCC1* genes; four different subfamilies are represented by different colors.

Cis-acting elements in gene promoters are crucial regions for initiating transcription at transcription factor-binding sites, which play an important role in regulating gene expression. The potential cis-acting elements on the promoter regions (2 kb upstream) of *TaRCC1s* were analyzed by PlantCARE to further explore their possible biological functions (details in **Supplementary Table S5**). Various potential cis-acting regulatory elements in the promoter regions of *TaRCC1* genes were predicted to be related to transcription, cell cycle, development, hormones, and response to stresses (**Figure 5**; **Supplementary Table S5**). All of the *TaRCC1* genes contained light-responsive elements. A total of 70 and 74*TaRCC1s* were detected with MeJA-responsive elements and ABA-responsive elements (ABRE), respectively. In addition, many elements were predicted to be involved in various abiotic stresses, such as drought, salt, cold, and light (**Figure 5**; **Supplementary Figure S1**; **Supplementary Table S5**).

### 3.6 Tissue-specific expression patterns of *TaRCC1s*

Using the available RNA-seq database of **(** International Wheat Genome Sequencing, C 2014 **)** obtained from the expVIP Wheat Expression Browser (http://www.wheat-expression.com/), the temporal and spatial expression patterns of 76 *TaRCC1* genes in five different tissues (root, stem, leaf, spike, and grain) (**Supplementary Table S6**) were visualized using the heatmap package of R software (**Supplementary Figure S2**). The expression levels of *TaRCC1s* varied significantly among different tissues. Some *TaRCC1s* from the same group showed similar expression patterns, while others indicated diverse expression patterns. For example, *TraesCS2B02G323100*, *TraesCS2A02G306400*, and *TraesCS2D02G304900* from TaRCC1 II were predominantly expressed in the root and stem, while the *TraesCS3A02G362800*, *TraesCS3B02G395200*, and *TraesCS3D02G356500* from *TaRCC1* II were most strongly expressed in the leaf, followed by the spike, root, and early stage of the stem. Similar expression patterns were observed for most homoeologous genes, although others presented diverse patterns. For instance, *TraesCS6A02G153100* and *TraesCS6D02G142500* were predominantly expressed in the root and stem, while the homoeologous gene *TraesCS6B02G180900* presented very low expression in the five tissues. It was worth noting that, in the *TaRCC1* I family, a total of four *TaRCC1* genes exhibited no expression in the five tissues.

### 3.7 Expression patterns of *TaRCC1s* under multiple stresses

**Figure 6:**
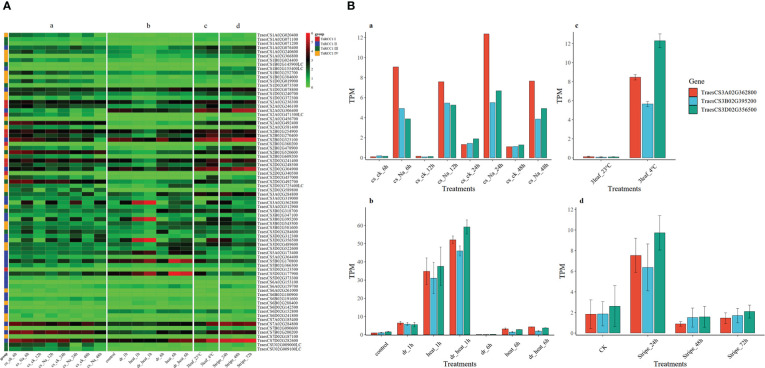
Expression profiles of *TaRCC1* genes under salt, drought, heat, cold and stripe rust pathogen stresses. **(A)** Expression heat map of 76 *TaRCC1* genes. Transcript-per-million-read (TPM) values of *TaRCC1* genes were transformed by log2(*x*+1) for visualization using the pheatmap package of R software. TPM values of the three genes were obtained from four published studies: (a) Zhang et al. (2016), (b) Liu et al. (2015), (c) Li et al. (2015), and (d) Zhang et al. (2014). **(B)** The expression histogram of *TaRCC1-3A/B/D* genes under salt, drought, heat, cold, and strip rust pathogen stresses. TPM values of the three genes were obtained from four public studies including: (a) the study of Zhang et al. (2016), (b) the study of Liu et al. (2015), (c) the study of Li et al. (2015), and (d) the study of Zhang et al. (2014).

The available RNA-seq data from four studies **(** Zhang et al., 2014; Li et al., 2015; Liu et al., 2015; Zhang et al., 2016 **)**, obtained from the expVIP Wheat Expression Browser (http://www.wheat-expression.com/) and Triticeae Multi-omics Center (http://202.194.139.32/expression/index.html), were used to study the expression of wheat *RCC1*s in response to salt, drought, heat, cold, and stripe rust stresses. The transcript-per-million-read (TPM) values of *TaRCC1* genes are presented in **Supplementary Table S7**; values were transformed by log_2_(*x*+1) and used for visualization with the pheatmap package of R software. The expression patterns of *TaRCC1* genes varied a lot under different stresses (**Figure 6A**). Although some homoeologous genes presented diverse expression patterns, most of them exhibited similar expression patterns. The homoeologous genes *TraesCS2A02G306400*, *TraesCS2B02G323100*, and *TraesCS2D02G304900* showed similar high expression trends under the four stresses and no significant differential expression under the different stresses, except for salt stress, indicating that these three genes might be induced by salt stress. The gene *TraesCS2A02G456700* exhibited lower expression only under salt stress, while the homoeologous genes *TraesCS2B02G478900* and *TraesCS2D02G457000* presented differential expression, especially under cold stress, suggesting these two genes might participate in the cold tolerance of wheat. The homoeologous genes *TraesCS3A02G362800*, *TraesCS3B02G395200*, and *TraesCS3D02G356500* showed similar and significant differential expression under the five different stresses. Under salt treatments at 6, 12, 24, and 48 h, the expression level of the three genes were higher than in the control; with drought and heat treatments, the three genes showed significantly higher expression under drought and/or heat treatments for 1 h, then decreasing after treatment for 6 h. Similar trends were detected under cold (4°C) and stripe rust pathogen stresses (**Figure 6B**). The results indicated that these three genes might be the key genes that participated in the early stress responses of wheat under stress, and might alleviate the stress injury of plants.

### 3.8 *Myb-7B/7D* transcription factor genes were predicted to regulate *RCC1-3A/B/D*

**Figure 7:**
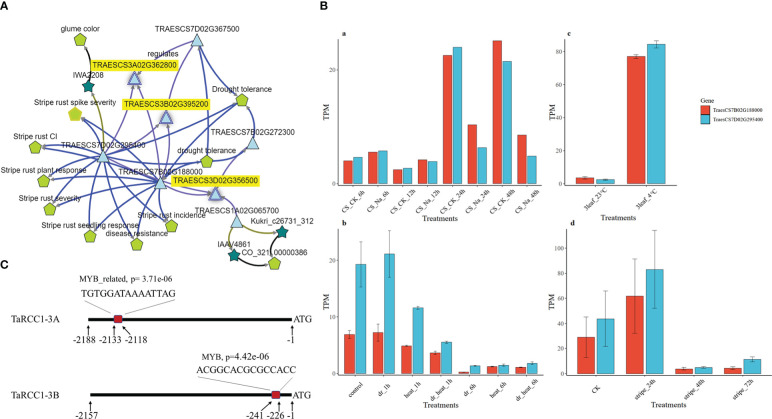
The Myb-7B/7D genes were predicted to regulate *RCC1-3A/B/D*. **(A)** Gene-evidence networks extracted from the wheat knowledge network for three *TaRCC1* genes through KnetMiner. **(B)** Expression histogram of *Myb-7B/7D* genes under salt, drought, heat, cold, and stripe rust pathogen stresses. Transcript-per-million-read (TPM) values of the three genes were obtained from four published studies: (a) Zhang et al. (2016), (b) Liu et al. (2015), (c) Li et al. (2015), and (d) Zhang et al. (2014). **(C)** The predicted Myb-binding sites upstream of the transcription start site of *TaRCC1-3A/B*.

The gene-evidence networks extracted from the knowledge network of wheat through KnetMiner showed several transcription factors for the three *TaRCC1* genes (**Figure 7A**). Five genes were identified as candidates for participating in the regulation of the three *TaRCC1s*, among which *Myb-7B* (*TraesCS7B02G188000)* and *Myb-7D (TraesCS7D02G295400)* were associated with the regulation of all three *TaRCC1s* (*RCC1-3A*, *RCC1-3B*, and *RCC1-3D*), suggesting that *Myb-7B* and *Myb-7D* might be the regulators of the three *TaRCC1s. Myb-7B* and *Myb-7D* encode two Myb-like transcription factors, which were related to the terms stripe rust response and drought tolerance in the KnetMiner knowledge network (**Figure 7A**). The expression patterns of *Myb-7B* and *Myb-7D* under multiple stresses, obtained from the expVIP Wheat Expression Browser (http://www.wheat-expression.com/) and the Triticeae Multi-omics Center (http://202.194.139.32/expression/index.html), showed similar and significant differential expression under different treatments of the five stresses. For instance, the expression level of the two *Myb* genes was higher than in the control for salt treatments at 6 h and 12 h and lower than in the control at 24 and 48 h. Under cold (4°C) and stripe rust pathogen stresses, the genes showed a similar gene expression pattern. Under drought and heat treatments, the *Myb-7B/D* showed reduced expression under dr_6h, heat_6h, and dr_heat_6h treatments, compared with the control (**Figure 7B**). Myb-binding sites on the promoters of *TaRCC1-3A*, *TaRCC1-3B*, and *TaRCC1-3D* were predicted using the PlantRegMap software (http://plantregmap.gao-lab.org/binding_site_prediction.php). A potential Myb-binding site was found in the promoters of *TaRCC1-3A* (−2119 to −2133) and *TaRCC1-3B* (−227 to −241), but no predicted Myb-binding sites were detected immediately upstream of the transcription start site of *TaRCC1-3D*, indicating that *TaRCC1-3A/B* are most likely directly regulated by the above Myb-like transcription factor, while *TaRCC1-3D* might be indirectly regulated (**Figure 7C**).

### 3.9 Subcellular localization of TaRCC1-3A/B/D and Myb-7B/D proteins

**Figure 8:**
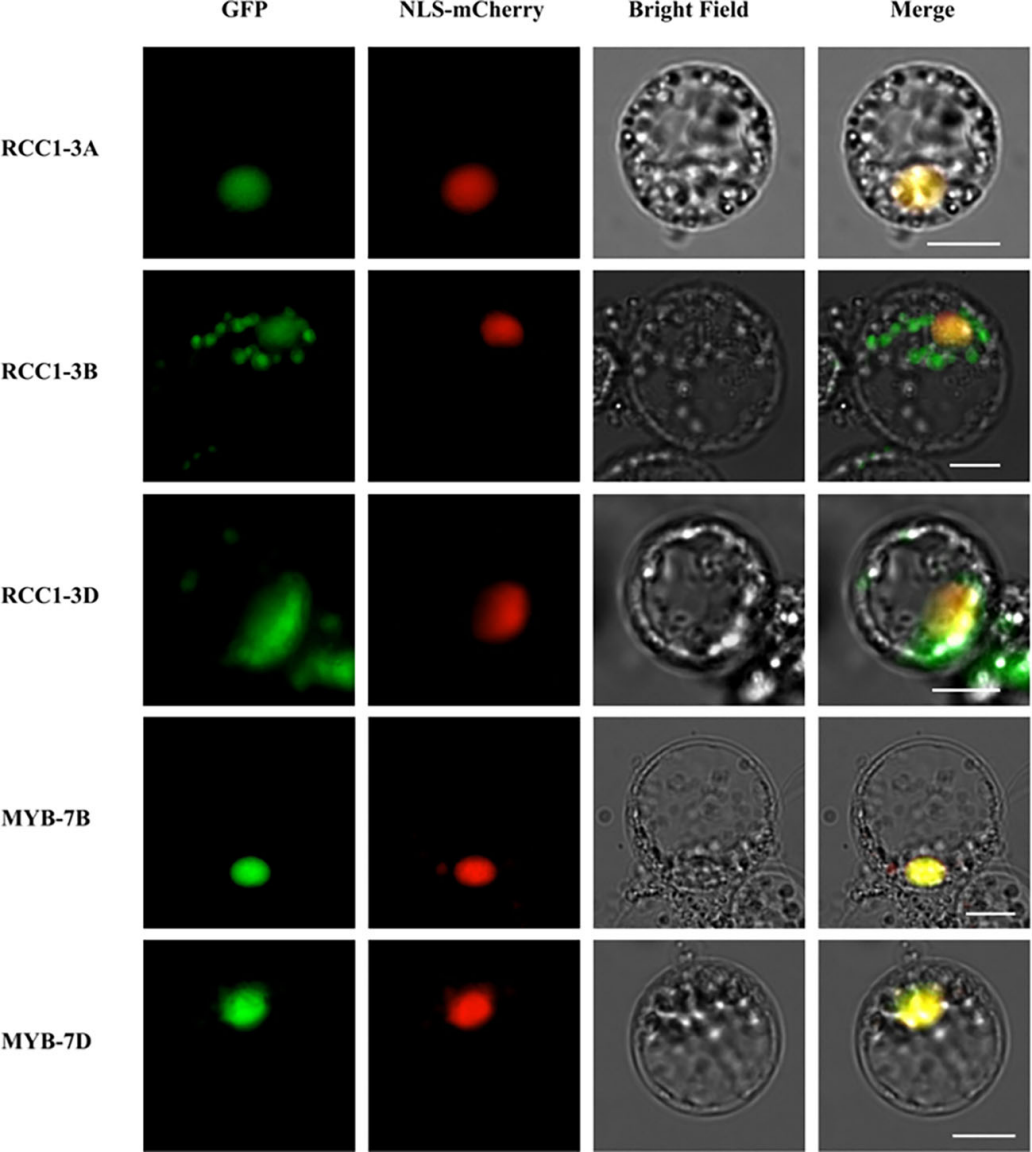
Subcellular localization of TaRCC1-3A (TraesCS3A02G362800), TaRCC1-3B (TraesCS3B02G395200), TaRCC1-3D (TraesCS3D02G356500), Myb-7B (TraesCS7B02G188000), and Myb-7D (TraesCS7D02G295400) proteins. TaRCC1-3A, TaRCC1-3B, TaRCC1-3D, Myb-7B, and Myb-7D were fused with GFP and co-expressed with the nuclear localization signal marker (NLS-mCherry) in wheat protoplasts. Scale bar = 20 μm.

In *Arabidopsis*, RCC1 family proteins, such as UVR8 and TCF1, are located in the nucleus. Similarly, subcellular localization prediction suggested that all of the TaRCC1 family proteins are located in the nucleus. Our experiments to investigate the subcellular localization of the three TaRCC1 proteins (TaRCC1-3A (TraesCS3A02G362800), TaRCC1-3B (TraesCS3B02G395200) and TaRCC1-3D (TraesCS3D02G356500) in wheat protoplasts confirmed the results as predicted and showed that these proteins are located in the nucleus (**Figure 8**). The subcellular localization of the Myb-7B/D proteins showed that these two Myb proteins are also located in the nucleus (**Figure 8**). The primers used are listed in **Supplementary Table S8**.

### 3.10 Expression patterns *via* qRT-PCR of *TaRCC1-3A/B/D* and *Myb-7B/D* in response to salt, cold, and drought stresses

**Figure 9:**
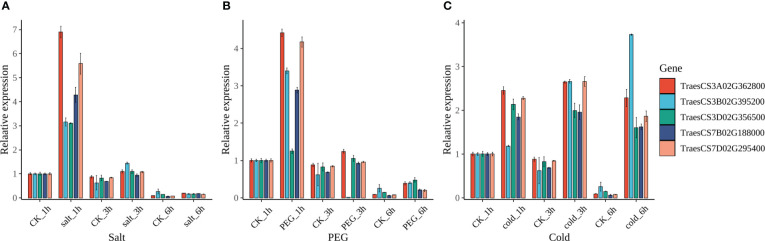
Expression pattern by qRT-PCR of *RCC1-3A*, *RCC1-3B*, *RCC1-3D*, *Myb-7B*, and *Myb-7D* under salt, drought, and cold stresses. **(A)** Salt stress, salt_1h/3h/6h: 1 h/3 h/6 h after watering with 1/2 MS liquid medium containing 200 mM NaCl. **(B)** Drought stress, PEG_1h/3h/6h: 1 h/3 h/6 h after watering with 1/2 MS liquid medium containing 20% PEG4000. **(C)** Cold stress, cold_1h/3h/6h: 1 h/3 h/6 h after watering with 1/2 MS liquid medium at 4°C. CK_1h/3h/6h in all three stress: 1 h/3 h/6 h after watering with 1/2 MS liquid medium at room temperature (control). Error bars represent the standard deviation of three biological replicates.

From the available RNA-seq data of several studies, the expression patterns of *TaRCC1s* in different tissues and multiple stresses had been analyzed, as described above (**Supplementary Figure S2**; **Figure 6**). The expression levels of *TaRCC1s* varied significantly in different tissues and under multiple stresses; several *TaRCC1s* were induced by different stresses. Homoeologous genes *TraesCS3A02G362800*, *TraesCS3B02G395200*, and *TraesCS3D02G356500* responded to five stresses (salt, drought, heat, cold, and stripe rust pathogen). We used qRT-PCR to verify the expression patterns of the three *TaRCC1s* in response to salt, cold, and drought stresses (**Figure 9**). Overall, these three *TaRCC1s* were induced by almost all the treatments, showing similar patterns to the RNA-seq results mentioned above. The expression of *Myb-7B* and *Myb-7D* was analyzed by qRT-PCR, and they shared a similar gene expression pattern to *RCC1-3A*, *RCC1-3B*, and *RCC1-3D* under salt, drought, and cold treatment (**Figure 9**), further suggesting that *Myb-7B* and *Myb-7D* might be regulators of the three *TaRCC1s* above. The qRT-PCR primers for *RCC1-3A*, *RCC1-3B*, *RCC1-3D*, *Myb-7B*, and *Myb-7D* are listed in **Supplementary Table S9**.

## 4 Discussion

The *RCC1* gene family is important in the functioning of the cell cycle. RCC1-like domains have been identified in a variety of proteins that mediate diverse biological processes **(** Hadjebi et al., 2008 **)**. Plant RCC1 proteins can be classified into two major groups, one consisting of six or seven RCC1 repeat units, similar to human RCC1, and the other composed of multi-domains, including the RCC1 repeat domain **(** Kuhn et al., 2011 **)**. In plants, however, the role of the *RCC1* family genes is still unknown. UVR8 and TCF1 in *Arabidopsis* belonging to the single domain RCC1 protein have been found to be involved in the regulation of signal cascades, such as UV-B and cold-induced signaling pathways **(** Heijde and Ulm, 2012; Li et al., 2013; Tilbrook et al., 2013; Jenkins, 2014; Ji et al.,2015 **)**. Wheat is the world’s most cultivated crop and an important staple food for humans, accounting for one-fifth of calories consumed **(** Abhinandan et al., 2018 **)**. The release of a high-quality wheat reference genome has enabled the rapid and systematic study of the function of wheat genes to develop. Sequencing projects provide an opportunity for the isolation of gene families using a genome-wide scan. In wheat, there has been no comprehensive study focusing on the *RCC1* genes, therefore, in this study, a comprehensive analysis of the *TaRCC1* genes, including studies of phylogenetic relationships, gene structure, conserved motifs, chromosomal location, and expression profiles in different tissues, was performed to characterize the gene family in bread wheat. We first isolated 144 *RCC1* genes, including 76 *TaRCC1s*, 49 *TdRCC1s*, and 24 *AetRCC1s* in wheat, emmer wheat, and *Aegilops tauschii*, respectively, identified from the fully annotated reference genomes. Phylogenetic analysis and synteny analysis showed that the *RCC1* genes were clustered into four subfamilies (named RCC1 I–IV). Most *TdRCC1s* (except *TRIDC3AG069370.1*) and all the *AeRCC1s* were high-collinear with the *TaRCC1s*. The collinear *RCC1* genes of the three species were clustered together in one clade (**Figures 2**, **3**), and the number of *RCC1s* of the three species showed an approximate 3:2:1 ratio. Because of the collinearity among the A, B, and D sub-genomes of wheat, most of the 76 *TaRCC1* genes identified were triplet genes (**Figure 3**). These results indicated that the *RCC1* genes are evolutionarily conserved in bread wheat, emmer wheat, and *Aegilops tauschii*.

Based on phylogenetic and gene structure analyses, the 76 *TaRCC1s* were clustered into four subfamilies (named *TaRCC1* I–IV). The *TaRCC1* I, II, and III subfamilies contained 10, 27, and 12 genes, which contained only RCC1 domain repeats. The remaining 27 *TaRCC1s* of *TaRCC1* IV contained multiple domains, including RCC1 domain repeats and PH or BRX domains (**Figure 4C**). These findings indicated that two different mechanisms might regulate genes in the *TaRCC1* family. It appears that most *TaRCC1* genes in a subfamily share a similar exon–intron structure, motif, and domain composition (**Figure 4**), indicating that the evolution might not only affect gene function but also gene structure **(** Babenko et al., 2004; Roy and Penny, 2007 **)**.

Analysis of the cis-acting regulatory elements in the promoter regions of *TaRCC1* genes showed that *TaRCC1s* might be involved in the regulation of various biological processes, and several cis-acting regulatory elements were especially related to responses to hormones and stresses (**Figure 5**; **Supplementary Table S5**). Thus, we can speculate that the wheat *RCC1* genes participate in specific signaling pathways that regulate growth, development, and defensive responses.

According to the publicly available transcriptome data of several studies, the expression profiles of *TaRCC1* genes in wheat varied among different tissues and developmental periods, and the *TaRCC1s* showed different expression patterns under different stresses, namely salt, drought, heat, cold, and stripe rust. Three homologous *TaRCC1s* (*TraesCS3A02G362800*, *TraesCS3B02G395200*, and *TraesCS3D02G356500*) were proved to respond to all five different stresses; the genes were induced by almost all the treatments, suggesting that they might participate in regulating the plant responses to numerous stresses. The RCC1 proteins in plants have been implicated in regulating gene expression *via* epigenetic mechanisms **(** Ji et al., 2015 **;** Ji et al., 2019 **;** Jiang et al., 2021 **;** Sun et al., 2022 **)**. Therefore, we determined whether the three TaRCC1 proteins above were located in the nucleus to investigate the possibility of their involvement in the regulation of downstream gene expression. Our results demonstrated that RCC1-3A, RCC1-3B, and RCC1-3D are all nuclear-localized proteins (**Figure 9**). At the same time, the green fluorescent protein (GFP) signals in wheat protoplasts were also obtained outside the nucleus. These results revealed the ability of RCC1-3A, RCC1-3B, and RCC1-3D proteins to migrate within cells, as has been reported for the UV-B receptor UVR8 **(** Kaiserli and Jenkins, 2007 **;** Yin et al., 2016 **)**. Moreover, two Myb transcription factor genes (*Myb-7B* and *Myb-7D*) that co-expressed with *RCC1-3A*, *RCC1-3B*, and *RCC1-3D* were identified by co-expression and bioinformatics analysis (**Figure 7C**), which suggested that Myb-7B and Myb-7D might bind to the promoters of *RCC1-3A/3B* and upregulate their expression in response to abiotic stresses. The roles of *RCC1-3A/3B/3D*, and their interaction with Myb-7B/D, need to be investigated further by mechanistic studies, for example using transgenic and yeast single hybrid experiments.

## 5 Conclusions

From the fully annotated reference genomes, 149 *RCC1* genes comprising 76 *TaRCC1s*, 49 *TdRCC1s*, and 24 *AetRCC1s* were identified in wheat, emmer wheat, and *Aegilops tauschii*, respectively. The 76 *TaRCC1s* in wheat were comprehensively analyzed in terms of gene structure, chromosome distribution, conserved domains, collinearity, phylogenetic relationship, and expression patterns in different tissues and in response to stresses. The expression patterns of 76 *TaRCC1s* in wheat under various stresses were further analyzed: qRT-PCR verified that *RCC1-3A* (*TraesCS3A02G362800*), *RCC1-3B* (*TraesCS3B02G395200*), and *RCC1-3D* (*TraesCS3D02G35650*) were significantly induced by salt, cold, and drought stresses. Co-expression network analysis and binding site predictions suggested that transcription factors encoded by *Myb-7B* (*TraesCS7B02G188000)* and *Myb-7D (TraesCS7D02G295400)* bind to the promoter of RCC1-3A/3B and upregulate gene expression in response to abiotic stresses in wheat. Our results provide valuable reference data for further study of RCC1 genes in wheat.

## Data availability statement

The datasets presented in this study can be found in online repositories. The names of the repository/repositories and accession number(s) can be found in the article/**Supplementary Material**.

## Author contributions

Conceptualization: XA and CS. Methodology: XA and CS. Software: WL. Validation: XA, WL, and TL. Formal analysis: XA. Investigation: XL. Resources: XA. Data curation: XA. Writing–original draft preparation: XA and LZ. Writing—review and editing: XA, CC, SZ, and CS. Visualization: XA. Supervision: XA. Project administration: XA. Funding acquisition: XA and CS. All authors contributed to the article and approved the submitted version.
