# Supplementary material for: Genome-wide identification and expression analysis of the regulator of chromosome condensation 1 gene family in wheat (Triticum aestivum L.)
Source: Front Plant Sci. 2023 Feb 24;14:1124905. doi: 10.3389/fpls.2023.1124905 (PMC9998523; doi:10.3389/fpls.2023.1124905)
Supplement: Supplementary file 2 [file DataSheet_1.docx]

Supplementary Figures


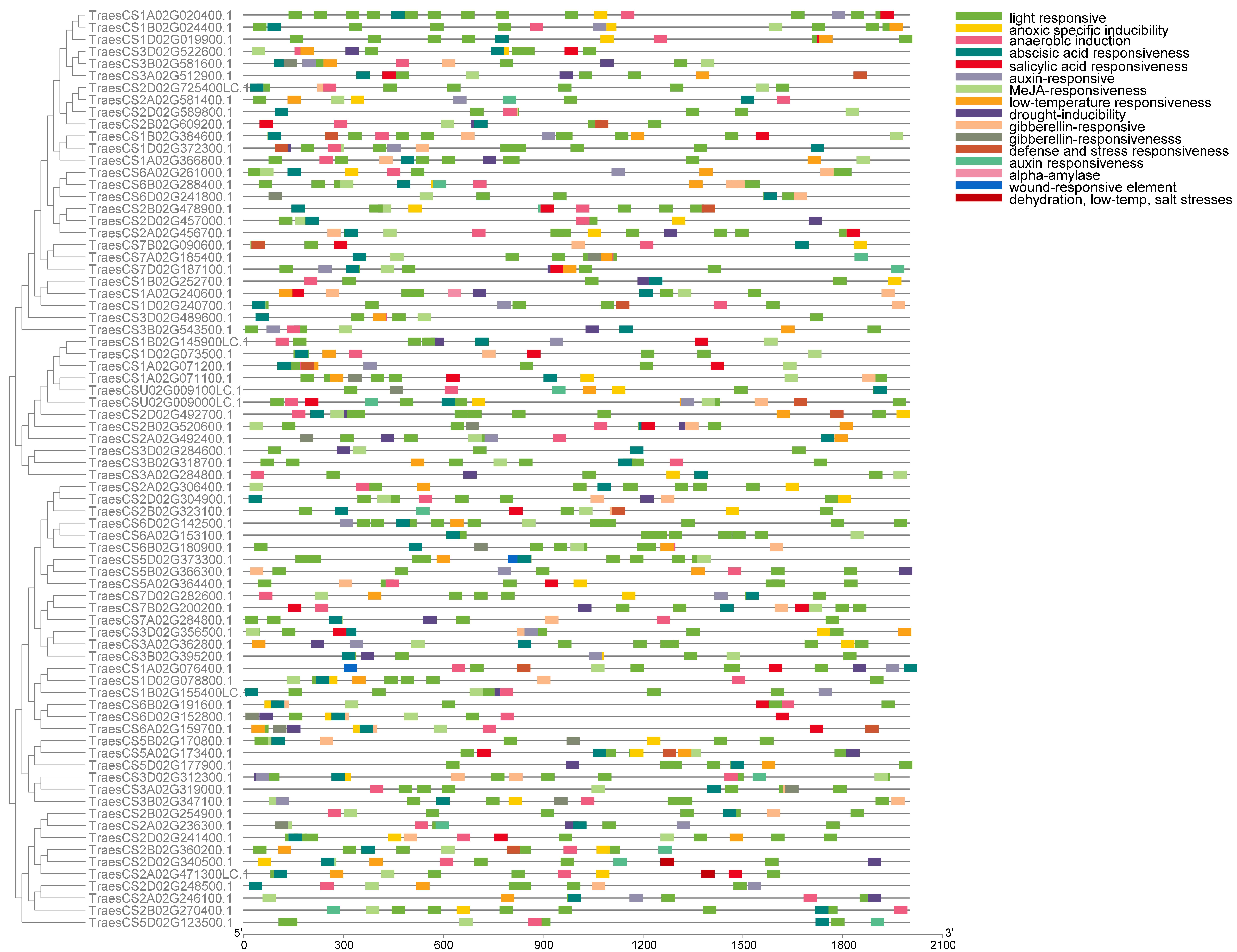


**Supplementary Figure 1**. Cis-acting regulatory elements related to hormone and abiotic stress responses in the promoters of TaRCC1s. The cis-acting regulatory elements are shown with different colored boxes.


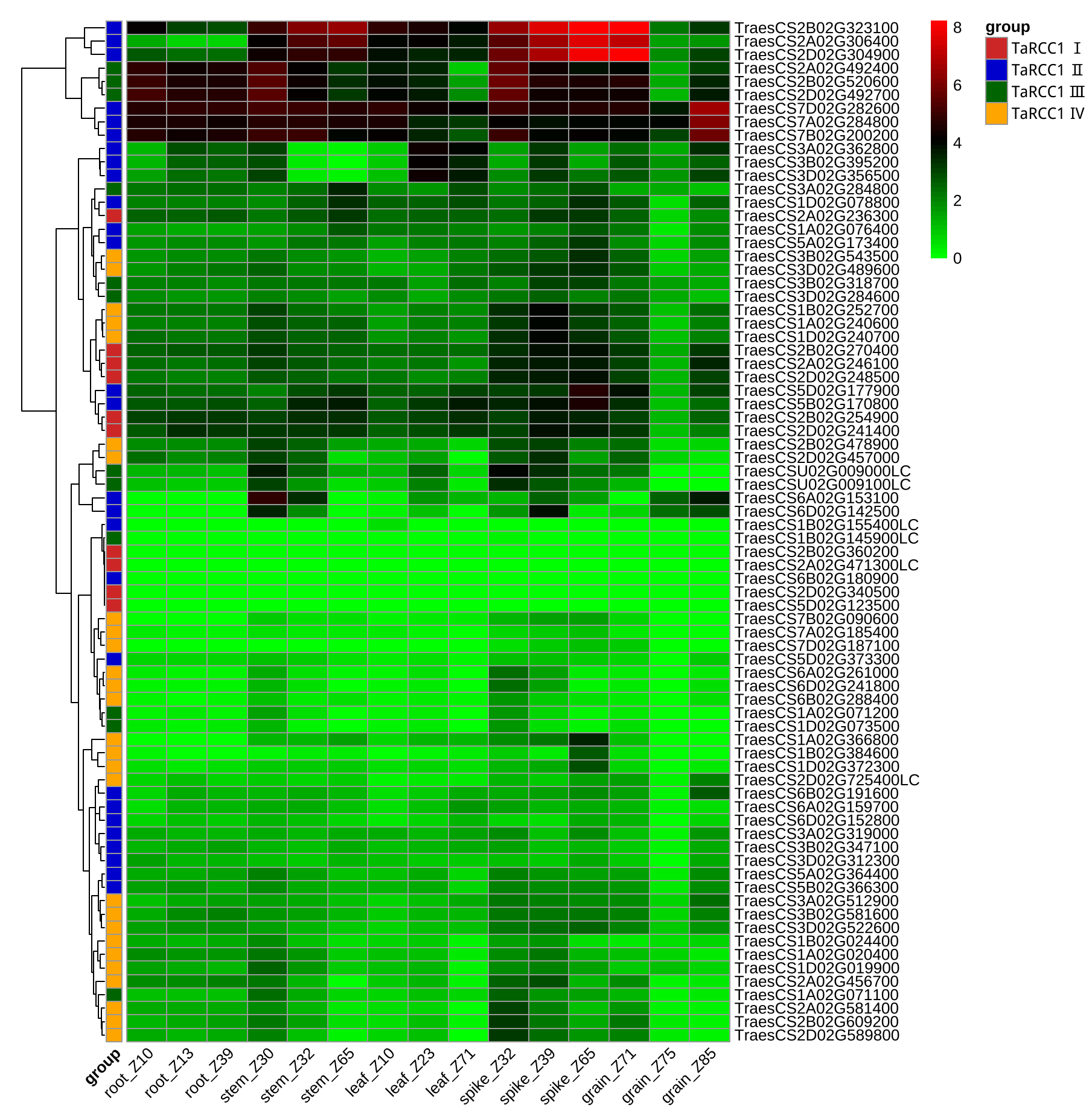


**Supplementary Figure 2**. Expression profiles of *TaRCC1*s in root, stem, leaf, spike, and grain. The heatmap was constructed using the heatmap package of R software, and the transcripts per million reads (TPM) values of *TaRCC1* genes were transformed by log_2_(x+1). The red and green colors represent higher and lower relative abundance of the transcript, respectively. root_Z10: root at seeding stage; root_Z13: root at three-leaf stage; root_Z39: root at flag leaf stage; stem_Z30: stem at 1 cm spike stage; stem_Z32: stem at two nodes detectable stage; stem_Z65: stem at anthesis stage; leaf_Z10: leaf at seedling stage; leaf_Z23: leaf at tillering stage; leaf_Z71: leaf at 2 d post-anthesis (dpa) stage; spike_Z32: spike at two nodes detectable stage; spike_Z39: spike at flag leaf stage; spike_Z65: spike at anthesis stage; grain_Z71: grain at 2 dpa stage; grain_Z75: grain at 14 dpa stage; grain_Z85: grain at 30 dpa stage.
